# Reducing regional health inequality: a sub-national distributional cost-effectiveness analysis of community-based treatment of childhood pneumonia in Ethiopia

**DOI:** 10.1186/s12939-020-01328-8

**Published:** 2021-01-06

**Authors:** Maria Olsen, Ole F. Norheim, Solomon Tessema Memirie

**Affiliations:** 1 Bergen Centre for Ethics and Priority Setting, Department of Global Public Health and Primary Care, University of Bergen, Bergen, Norway; 2 Department of Pediatrics and Child health, College of Health Sciences, Addis Ababa University, Addis Ababa, Ethiopia

**Keywords:** Global health, Health systems, Priority setting, Justice, Equity, Geographic equality, Cost-effectiveness, Pneumonia

## Abstract

**Background:**

Increasing the coverage of community-based treatment of childhood pneumonia (CCM) is part of the strategy to improve child survival, increase life-expectancy at birth and promote equity in Ethiopia. However, full coverage of CCM has not been reached in any regions of the country. There are no sub-national cost-effectiveness analyses available to inform decision makers on the most equitable scale up strategy.

**Objectives:**

Our first objective is to estimate the sub-national cost-effectiveness and the interindividual inequality impacts of scaling up CCM coverages to 90% in each region. Our second objective is to explore the costs, health effects, and geographical inequality impacts associated with three scale-up scenarios promoting different policy-aims: maximizing health, reducing geographical inequalities, and achieving 90% universal coverage.

**Methods:**

We used Markov modelling to estimate the sub-national cost-effectiveness of CCM in each region. All data were collected through literature review and adjusted to the region-specific proportions of the rural population. Health effects were modeled as life years gained and under-five deaths averted. Interindividual and geographical inequality impacts were measured by the GINI index applied to health. In scenario analysis we explored three different scale-up strategies: 1) maximizing health by prioritizing the regions where the intervention was the most cost-effective, 2) reducing geographical inequalities by prioritizing the regions with high baseline under-five mortality rate (U5MR), and 3) universal upscaling to 90% coverage in all the regions.

**Results:**

The regional incremental-cost effectiveness ratio (ICER) of scaling up the intervention coverage varied from 26 USD per life year gained in Addis to 199 USD per life year gained in the Southern Nations, Nationalities, and Peoples’ region. Universal upscaling of CCM in all regions would cost about 1.3 billion USD and prevent about 90,000 under-five deaths. This is less than 15,000 USD per life saved and translates to an increase in life expectancy at birth of 1.6 years across Ethiopia. In scenario analysis, we found that prioritizing regions with high U5MR is effective in reducing geographical inequalities, although at the cost of fewer lives saved as compared to the health maximizing strategy.

**Conclusions:**

Our model results illustrate a trade-off between maximizing health and reducing health inequalities, two common policy-aims in low-income settings.

## Introduction

Universal health coverage (UHC) is achieved when the entire population has access to essential medical services and can access these services without being exposed to financial hardship [1]. Achieving universal health coverage is high on the agenda of the 2030 sustainable development goals and tightly interlinked with all the focus areas on poverty, inequality and health. However, health resources are limited and there is an inevitable need to set priorities when progressing towards universal health coverage. Policy makers allocating scarce resources need evidence on expected costs and benefits associated with different scale-up strategies for essential health interventions.

Ethiopia has achieved considerable improvements in population health and child survival over the past decade. However, many preventable deaths are still occurring, and some regions seem to be left behind in the progress that has been made. In 2016, the under-five mortality rate ranged from 39 deaths per 1000 live births in Addis Ababa to 125 deaths per 1000 live births in Afar [2]. Pneumonia was among the three major causes of under-five mortality throughout Ethiopia. Effective treatment is available, but the coverage remains low. Scaling up the coverage of community-based treatment for childhood pneumonia (CCM) through the Health Extension Program could be an effective strategy to further reduce the under-five mortality rate (U5MR) and promote equity.

The Health Extension Program was launched by the government in 2004 with the aim of increasing coverage of primary health care services in Ethiopia. The Health Extension Program consists of local, government salaried women who do preventive work and provide basic curative services in their assigned *kebele* (community). Two health extension workers are typically stationed at each local health posts serving a population of 5000 people on average. Integrated community case management (iCCM) and community-based treatment of pneumonia (CCM) in children less than 5 years of age were included as part of the essential services provided by the health extension workers in 2010. However, only 29.2% of rural children and 59.1% of urban children with symptoms of pneumonia sought treatment in 2016 [2].

Despite efforts to reach rural and poor population with high impact interventions through the Health Extension Program, evidence suggests that inequalities in important child health indicators have increased [3]. Distance to the nearest health facility and to the region of residence are two important geographical factors that influence both health service utilization and child health outcomes [4, 5]. In general, poor and rural populations are often deprived of health compared to rich and urban populations. Moreover, there are currently substantial geographical inequalities in health service coverage, life expectancy at birth and child health outcomes between the major regions of Ethiopia [3]. These geographical health inequalities are avoidable and most often associated with geographical inequalities in the distribution of other resources, such as income.

When progressing towards UHC, policy makers need to consider both the inequality impacts and the cost-effectiveness of prioritizing different strategies. Previous studies have already shown that pneumonia treatment is pro-poor and provides protection from financial risk [6, 7]. It has been shown previously that scaling up coverage of pneumonia treatment would be cost-effective at a national level [6, 8]. However, to our knowledge, no studies on sub-national cost-effectiveness of pneumonia treatment have yet been published, nor have previous studies investigated the impacts of scaling up treatment coverage on geographical inequalities.

The regions of Ethiopia are diverse in terms of influential factors such as available infrastructure, wealth, local epidemiology, background mortality and baseline coverage of pneumonia treatment. These factors are likely to affect both the costs and the expected benefits of providing pneumonia treatment at the community level, and we believe that data on regional cost-effectiveness would inform policy planning more effectively than national averages alone [9]. Our first aim is to model the sub-national cost-effectiveness and the inequality impacts of scaling up coverage of community-based treatment of childhood pneumonia (CCM) in each region. Our second aim is to explore the costs, health effects and geographical inequality impacts of three scale-up strategies promoting different policy aims: decreasing geographical inequalities in life expectancy and child survival, maximizing health, and universal scale-up.

## Methods

We used Markov modeling in the software program TreeAge to model health and economic impacts of scaling up coverage of CCM from baseline to a target coverage of 90% in each of Ethiopia’s eleven major regions. Each regional model was populated with 2016 region-specific data on cost per treatment, incidence of childhood pneumonia, background mortality, and baseline coverage of the intervention. Untreated case fatality rate and treatment effect were assumed to be similar in all the regions. Region-specific inputs are displayed in Table 1 and fixed model inputs are displayed in Table 2. We assumed that all treatments were provided to outpatients under universal public coverage.

**Table 1:** region specific model inputs (data source: [2])

Region	Background infant mortality^a^ per 1000	Background child mortality^b^ per 1000	Cost per treatment (USD)	Incidence of pneumonia^c^	Baseline coverage (2016)
Afar	76.7	11.5	247.8	0.244	44 %
Beni-S.	60.2	9.2	271.3	0.102	29 %
Somali	50.5	4.3	271.3	0.119	32 %
Dire Dawa	16.4	1.7	124.8	0.221	50 %
Gambela	60.2	9.2	124.8	0.198	29 %
SNNP	64.9	7.0	288	0.385	43 %
Amhara	57.3	4.1	277.7	0.453	29 %
Oromia	56.6	3.9	281.6	0.419	26.4 %
Harari	52.9	7.8	163.9	0.04	45 % ^a^
Tigray	57.8	5.6	255.1	0.436	34 %
Addis	30.0	5.0	45	0.153	59 %
National	**48.5**	**5.1**	**262.1**	**0.397**	**33 %**
Urban					59.1 %
Rural					29.2 %

^a^Infant mortality is the risk of dying before reaching the age of one

^b^Child mortality is the yearly risk of dying in the age group 1-5

^c^Calculated by assuming 4.6 days mean duration of disease [2, 10]

**Table 2:** Fixed model inputs

Model parameters		References/assumptions
Untreated case fatality rate (CFR)	0.0351	[11]
Treatment efficacy in reducing CFR	0.70	[12, 13]
Cycles	120	Lifetime analysis
Discounting of costs	0.03	[14]
Discounting of effects	0.03	[14]
Target coverage	0.90	Per WHO definition of UHC
2016 GDP per capita	713 USD	[15]

### Markov modeling

**Fig. 1:**
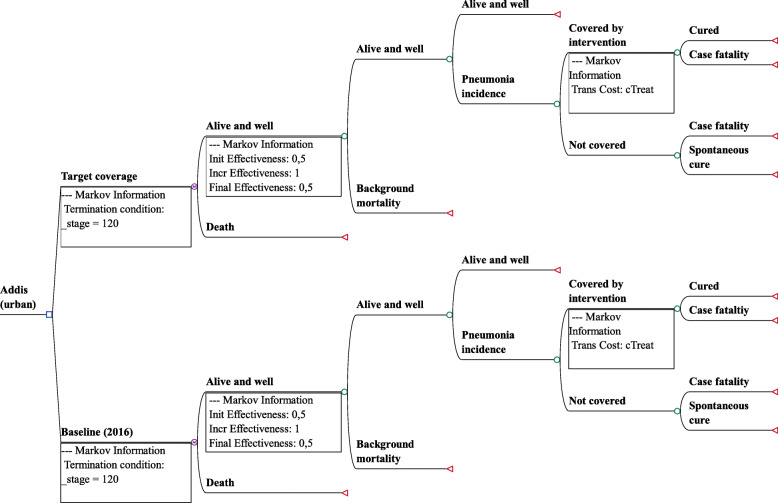
Markov model

**Fig. 2:**
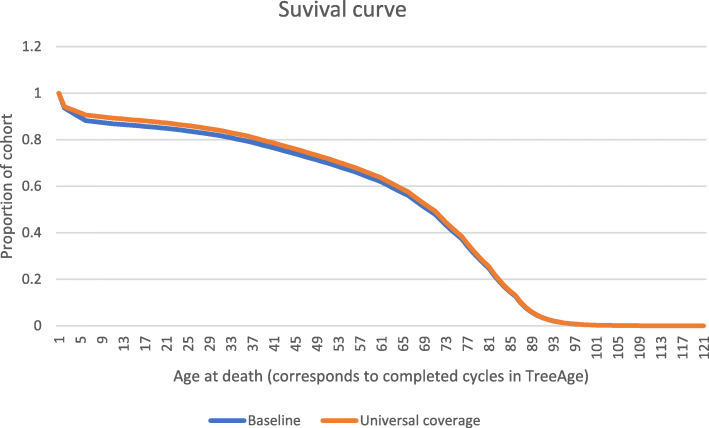
Population survival curves under baseline and universal coverage

Figure 1 provides an overview of our Markov model. The model has two arms labelled *baseline* and *target coverage*. The branches within the arms represent the probabilities that individuals within the modeled 2016 birth cohort would progress through the health events labeled at each branch. The CCM intervention is present during only the first five cycles. At the end of each cycle, one proportion of the cohort moves back to the “Alive and well” status, whereas the other proportion moves to the “Death” status. Those who returned to the “Death” status are removed from the model, whereas each return to the “Alive and well” status is counted as one life year completed (illustrated by the survival curves in Fig. 2). We extrapolated results by running the model for a total of 120 cycles until everyone in the model cohort was dead. Since the model starts at age zero and runs for 120 cycles, output adds up to life expectancy. Model inputs were specific for each region, but the structure of the models did not change.

### Model inputs on incidence of pneumonia

Data on prevalence of pneumonia, under-five mortality rates by all causes and baseline coverage in each region were collected from the Demographic and Health Survey 2016 (DHS 2016). The DHS 2016 estimated the prevalence of pneumonia by asking mothers of children under 5 years of age whether their child had experienced clinical symptoms of pneumonia during the 2 weeks prior to the interview. Clinical symptoms are defined as cough accompanied by short, rapid breathing that was chest-related, and/or difficult breathing that was chest-related [2]. In this survey, baseline treatment coverage was estimated as the percentage of children with symptoms of pneumonia who received clinical examinations and oral antibiotics from a health professional.

We used the following formula to estimate the annual incidence of pneumonia among children below the age of five:

Incidence = prevalence/duration of disease [16]

The estimated incidence of pneumonia varied from 0.453 cases per year in the Amhara region to 0.040 cases per year in the Harari region (Table 2). Our calculations were comparable to estimates of incidence of childhood pneumonia in comparable settings [11].

### Model inputs on background mortality

Age-specific mortality rates among adults affect the incremental life years gained by reducing U5MRs, and since U5MR is an acknowledged predictor of general population health, the large regional inequalities in U5MRs are likely to be reflected in mortality among older age groups. However, only life tables representing national averages were available for age-specific mortality rates among adults and children older than 5 years of age. Therefore, we modeled adult mortality rates based on the assumption that the U5MR is associated with mortality among older age groups of the same population. In practice, we selected Ethiopian abridged life tables from the 2015 UN world population prospect [17] and matched each region to a national life table in a time period with a similar U5MR as the one observed for the region in 2016 [18]. The life table from this time period was used as a proxy for adult mortality rates in that region.

There are no census data from 2016. Without taking into account the effect of still births, we used regional fertility rates and data on the total number of women in each region to yield a rough estimate of the number of births in 2016 [2, 19]. We used these estimates as size variables in the calculations of weighted averages of the effects observed in each region, the budget impact and the geographical Gini coefficients.

### Model inputs on the effectiveness and unit costs of CCM

Data on effectiveness of the treatment were collected from a previously published systematic review of studies assessing CCM of pneumonia in developing countries [12]. The review concluded that CCM on average reduces the case fatality rate of pneumonia in children less than 5 years of age by 70% (Table 2). We applied this as our input for treatment effects in all the regions.

Data on treatment costs were collected through literature review. We did not encounter any data on regional cost per treatment. However, previous studies indicate that there are significant differences in costs of providing community health services in different geographical contexts [9, 20]. The inputs for costs per treatment in each region were therefore adjusted for rural and urban residency.

One study from Kenya showed that it was 7.2 times more expensive to provide community health services in rural areas compared to urban areas [9]. We assumed that rural Ethiopia would observe a similar increase in cost per treatment compared to urban Ethiopia. We applied a cost per treatment provided for patients with urban residency of 45 USD [8, 21], and the cost per treatment provided for patients with rural residency was modelled to be 7.2 times more expensive (Table 1). However, most regions of Ethiopia have both rural and urban population [22]. The following formula was used to estimate average costs per treatment in each region: *Average cost per treatment* = (45 ∗ *x*) + (45 ∗ 7.2 ∗ *y*),where x is the proportion with urban residency while y is the proportion with rural residency.

The studies we relied on to estimate the costs per treatment costed the intervention from a providers’ perspective. Cost items classified as personnel costs, capital costs or supply costs were included. These were further divided into patient care costs and overhead costs [21]. In our Markov modeling, we did not include initial training of health personnel or capital costs. Costs were discounted at a 3% rate.

### Estimation of the intervention effects on health and health inequalities

We modeled effects of scaling up coverage of CCM as life expectancy gains for children less than 5 years of age. The children who recovered from pneumonia were assumed to continue to live with the same health risks as the overall population. We did not apply disability weights to the effect measure as we were primary interested in mortality reduction afforded by the intervention. Hence, the incremental effects of the intervention represent gains in life expectancy at birth. We half-cycle corrected and discounted the effects at a 3% rate. The incremental cost-effectiveness ratios were calculated by dividing incremental costs by incremental life years gained.

The Gini coefficient is a measure of inequality (here applied to health), represented by a number between 0 and 1 where 0 represents absolute equality in the distribution of a chosen variable, and 1 represents a situation in which all of the chosen variable belong to one individual. Gini coefficients can be used to describe inequalities in life expectancy between individuals or population groups [23]. We used the DASP extension of the STATA software to calculate the Gini coefficients quantifying inequalities in life expectancy between the regions and between individuals within each region. In our calculations of geographical inequalities, we applied model results on regional life expectancies at birth as the health variable, and estimated numbers for children born in 2016 as the size variables. Data from survival curves provided by the Markov models were applied as the size and health variables for calculation of interindividual health inequalities.

### Scale-up scenarios

In the regional scenario analysis, we explored three possible objectives: health maximization, reducing geographic inequality, and universal scale-up. The first two scenarios have lower costs and could be possible pathways to universal scale-up. We estimated incremental costs, reduction in national U5MR, incremental effects, and interindividual GINI impacts of 1) maximizing health by scaling up to 90% coverage in the six regions where the intervention is the most cost-effective, 2) reducing geographic inequality by scaling up to 90% coverage in the three regions with the highest under five mortality rates, and 3) universal scale-up to 90% coverage in all regions. For all scenarios, we estimated expected health impacts across Ethiopia by adding the weighted averages of the effects observed in each region.

## Results

**Fig. 3:**
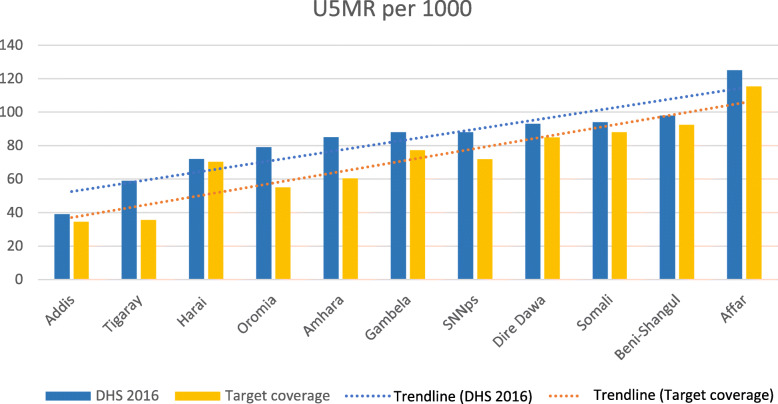
Under five mortality rates in each region at baseline and universal coverage. **. **Geographical GINI and the slope of inequality: The geographical GINI indexes are determined by the slope of inequality in Fig. 3 (dotted lines) and by the size of the regions. Since the size variable remains the same before and after scale-up, the geographical GINI index will increase from one scenario to another if the slope of the inequality increases, as illustrated in the comparison between baseline coverage and universal coverage. Conversely, the GINI index would decrease if the slope of inequality decreased

In total, scaling up coverage of CCM to 90% in all regions would decrease the Ethiopian U5MR from 67 to 52 deaths per 1000 live births. Figure 3 shows the impact on U5MR in each region.

Reduced U5MR translates as increased life expectancy. Scaling-up treatment coverage to 90% in all regions would increase the average life expectancy at birth from 63.18 to 64.73 years, a 1.55 years gain. At a regional level, the incremental effects of scaling up the intervention coverage to 90% varied from 0.13 life years gained in Harari to 1.93 life years gained in Oromia (Table 3). The highest incremental effects were observed in regions with a high incidence of pneumonia and low baseline coverage of the intervention.

**Table 3:** Life expectancy at target and baseline coverage

Region	Life expectancy at baseline coverage	Life expectancy at target coverage	Incremental effects (life expectancy gains)
Affar	52.13	52.82	0.69
Amhara	56.42	58.30	1.89
SNPS	56.95	58.19	1.24
Beni-Shangul	57.84	58.27	0.43
Gambela	57.85	58.68	0.83
Somali	57.95	58.41	0.46
Dire Dawa	58.86	59.48	0.62
Oromia	59.76	61.69	1.93
Tigray	61.04	62.84	1.80
Harari	62.74	62.87	0.13
Addis	70.69	71.09	0.40

Table 4 shows total annual costs of the intervention at target and baseline coverage in each region and in total. Universal scale-up of CCM would cost about 1.3 billion USD. The incremental costs of scaling up intervention coverage were highest in regions with a large rural population, low baseline coverage of the intervention and high incidence of pneumonia. The program costs increased further in regions with a large 2016 birth cohort, such as the SNNP region.

**Table 4:** Total and incremental costs (USD)

	Total cost at baseline coverage (USD)	Total cost at target coverage (USD)	Incremental costs (USD)
Addis	1,200,000	1,900,000	600,000
Tigaray	32,100,000	86,000,000	54,000,000
Harai	200,000	400,000	200,000
Amhara	138,400,000	434,100,000	295,700,000
Dire Dawa	1,100,000	1,900,000	900,000
Gambela	500,000	1,600,000	1,000,000
Somali	21,000,000	59,000,000	38,200,000
Beni-Shangul	1,800,000	5,600,000	3,900,000
Oromia	273,000,000	943,000,000	669,000,000
SNPS	219,500,000	463,500,000	244,000,000
Affar	12,000,000	24,700,000	12,700,000
**SUM**	700,800,000	2,022,000,000	1,321,000,000

**Fig. 4:**
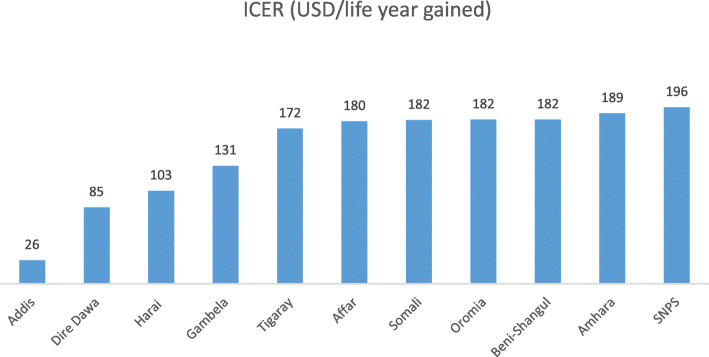
Incremental cost effectiveness ratio (ICER) in each region

The incremental cost-effectiveness ratio (ICER) of scaling up coverage ranged from 26.15 USD per life year gained in Addis to 195.80 USD per life year gained in SNNP region (Fig. 4).

Increasing the coverage of pneumonia treatment would decrease U5MR and alter the survival curves, translating as reduced interindividual inequalities. Table 5 shows the Gini coefficients for interindividual inequalities in life expectancy within each of the 11 regions at baseline and target coverage. Reducing U5MR by increasing coverage of CCM translates as decreased interindividual inequalities in all the regions.

**Table 5:** Gini coefficients for interindividual inequalities in each region

Region	Gini at baseline coverage	Gini at target coverage
Afar	0.228	0.225
Amhara	0.202	0.199
Ben-Shangul	0.210	0.207
Dire Dawa	0.203	0.199
Gambela	0.210	0.205
Harari	0.191	0.190
Oromia	0.212	0.198
SNPS	0.216	0.208
Somali	0.210	0.207
Tigaray	0.187	0.174
Addis	0.153	0.149

### Scenario analysis

Table 6 shows the incremental costs, the incremental life years gained, the decrease in U5MR and the interindividual and geographical Gini impacts of three possible scenarios: health maximization, decreasing geographical inequalities, and universal scale-up. Addis Ababa, Gambela, Dire Dawa, Harari and Tigaray were the regions in which the intervention was the most cost-effective, and Afar, Beni-Shangul and Somali were the regions with the highest baseline under five mortality rates.

**Table 6:** Scenario analysis

Scenario	Incremental cost (USD)	Cost per death averted (USD)	Life expectancy gain	Interindividual GINI	Geographical GINI
Baseline	–	–	–	0.186	0.018
Health maximization	56,800,000	13,407	0.071	0.185	0.019
Decreasing geographical inequalities	54,800,000	14,323	0.068	0.185	0.017
Scale-up to 90% in all regions	1,321,000,000	14,530	1.550	0.177	0.019

Universal scale-up to 90% coverage in all regions would substantially alter the survival curves and decrease interindividual inequalities in life expectancy (Table 6). The health maximizing strategy and the strategy prioritizing the worse off both yielded slight decreases in interindividual Gini with much lower incremental costs (Table 6). As shown *in* Table 6, only the targeted strategy to increase treatment coverage in the regions with the highest U5MR decreased regional inequalities in life expectancy. The other two strategies, health maximization and universal scale-up, both increased regional inequalities in life expectancy.

## Discussion

According to the final report of the WHO Consultative Group on Equity and Universal Health Coverage, cost-effectiveness, priority to the worse off, and financial risk protection are important concerns for evaluating which health care interventions should be ranked first in a universal health care package [24]. They also argue that efforts should be made to prevent underprivileged sub-populations from being left behind. Our results indicate that scaling up coverage of pneumonia treatment would be cost-effective in all the eleven major regions of Ethiopia, and that the intervention could reduce both interindividual and geographical inequalities in life expectancy. Moreover, making targeted efforts to scale-up the coverage of high priority interventions in underprivileged regions first would prevent groups of people disadvantaged by residence from being left behind.

Our results on incremental cost effectiveness ratio of scaling up pneumonia treatment coverage in the eleven major regions are comparable to those of published literature, in which we found incremental cost effectiveness ratios (ICERs) ranging from 26,6 to 208 USD per DALY averted [25, 26]. Geographical factors varying between the different study settings may account for the large dissimilarities found in previously published cost-effectiveness analysis. However, few previous studies have investigated subnational cost-effectiveness of community case management of pneumonia, nor the impacts of rural residence on costs per treatment.

There are several data limitations and weaknesses in this study. The first limitation is our estimation of costs per treatment in rural compared to urban areas. The study we relied on for these calculations was small and undertaken in Kenya which has a different strategy for delivery of community-based services [9]. However, we assumed that geographically varying factors identified in the Kenyan study, such as population density and attrition rates among local health workers, would be comparable to the Ethiopian context. In 2016, Berman et al. reported the costs of providing primary health care services as varying by a factor of more than five between the regions of Ethiopia [20]. Their results support our estimates of region-specific costs per treatment.

The second limitation is that we assumed the untreated case fatality rate of pneumonia was similar across all regions of Ethiopia. This may not be the case. Malnutrition, coinfection with HIV and low birth weight are some of the factors that influence the severity of pneumonia infections in children [27]. The prevalence of these risk factors varies between regions. Essentially, stronger evidence on sub-national cost-effectiveness would require more primary studies on how much geographical and regional factors influence case fatality rates and other key inputs for economic analysis.

We based our estimations of yearly incidence of pneumonia on data from the Demographic and Health survey 2016 [2]. Some of these estimations are high when compared to results from other studies [28]. There are several possible explanations. Firstly, the DHS data could have been collected during high season of the disease. Secondly, some of the children identified as having had pneumonia might have had other medical explanations of their symptoms, such as common cold. In the survey, pneumonia was not confirmed as the cause of symptoms by any biomedical tests.

A third limitation is that we did not include costs of demand generation, although evidence suggests that demand side barriers are major causes of sustained low intervention coverage [29, 30]. To focus on demand generation would be especially appropriate in Ethiopia where community health workers have already been deployed throughout the country but the utilization of key health services remains low [30]. Strategies such as the health development army have been initiated, but to our knowledge there is yet no evidence available on its associated costs and effects.

The cost-effectiveness threshold is the upper ICER to be considered cost-effective within a given health budget. Investing in health interventions that are above this threshold would draw resources away from more cost-effective interventions and would reduce total population health [31]. If we adopt a cost-effectiveness threshold of 50% of the GDP per capita, as suggested by Woods et al., scaling up coverage of CCM to 90% would be cost effective in all the regions of Ethiopia. However, even the threshold of 50% of the GDP per capita could be too high. Woods et al. indicate that a cost-effectiveness threshold between 4 and 51% of GDP per capita could be more appropriate for low to middle income countries, such as Ethiopia [31].

Concerns about reducing inequalities may justify giving priority to investment in regions where the health interventions are proven less cost-effective than in other regions where the interventions are more cost-effective [24]. This trade-off depends on the decision makers’ level of aversion to inequality [32]. All the scale-up strategies we explored reduced interindividual inequality at a regional and national level. However, only the strategy prioritizing the worst-off regions reduced geographical inequalities. Since there are considerable inequalities in wealth between the Ethiopian regions, reducing geographical inequalities in U5MR could also indirectly reduce socioeconomic inequalities [3].

## Conclusion

Pneumonia is among the major causes of under-five mortality in Ethiopia and increasing treatment coverage effectively reduces the under-five mortality rate in all eleven regions. This translates to decreased interindividual inequalities and increased life expectancy at birth. Despite large regional variations in incremental cost-effectiveness ratios, our results indicate that pneumonia treatment remains cost-effective across Ethiopia. However, scaling up treatment coverage to 90% in all the regions simultaneously would require substantial resources and, in practice, the scale-up is more likely to happen gradually. Prioritizing the intervention scale-up in the regions with the highest baseline U5MRs would reduce geographical inequalities in life expectancy; however, this would save fewer lives as compared to the health maximizing strategy. Of course, several other scale-up strategies are possible. Our results highlight the challenging trade-off between two common policy aims for health budgeting in low-income countries with geographical diversity: heath maximization and inequality reduction.

## Data Availability

All supporting data, including population prospects, costing of the CCM intervention, medical trials of CCM, and epidemiological data from the Demographic and Health Survey 2016, are freely available online and referred to in the reference list.

## Abbreviations

UHC: Universal health coverage
CCM: Community case management
iCCM: Integrated community case management
U5MR: Under five mortality rate
